# Publisher Correction: Ellipticity dependence of high-harmonic generation in solids originating from coupled intraband and interband dynamics

**DOI:** 10.1038/s41467-017-01768-x

**Published:** 2017-11-30

**Authors:** Nicolas Tancogne-Dejean, Oliver D. Mücke, Franz X. Kärtner, Angel Rubio

**Affiliations:** 1 Max Planck Institute for the Structure and Dynamics of Matter, Luruper Chaussee 149, 22761 Hamburg, Germany; 2 European Theoretical Spectroscopy Facility (ETSF), Luruper Chaussee 149 22761 Hamburg, Germany; 3 Center for Free-Electron Laser Science CFEL, Deutsches Elektronen-Synchrotron DESY, Notkestraße 85, 22607 Hamburg, Germany; 4 The Hamburg Center for Ultrafast Imaging, Luruper Chaussee 149, 22761 Hamburg, Germany; 5 Physics Department, University of Hamburg, Luruper Chaussee 149, 22761 Hamburg, Germany

*Nature Communications* **8**:745 doi:10.1038/s41467-017-00764-5; Article published online: 29 Sep 2017

The published version of this Article contained an error in the second sentence of the fourth paragraph of the subheaded section “Ellipticity and helicity of the emitted harmonics”. The final exponent in this sentence should read: ^*in*π/2^. This has now been corrected in the PDF and HTML versions of the Article.

