# Supplementary material for: Ellipticity dependence of high-harmonic generation in solids originating from coupled intraband and interband dynamics
Source: Nat Commun. 2017 Sep 29;8:745. doi: 10.1038/s41467-017-00764-5 (PMC5622149; doi:10.1038/s41467-017-00764-5)
Supplement: Supplementary file 1 — Supplementary Information [file 41467_2017_764_MOESM1_ESM.pdf]

Supplementary note 1: Effect of the dynamical correlations on the HHG spectra of bulk magnesium oxide

We have investigated the effect of dynamical correlations on the HHG spectra of bulk MgO, following the approach of [1]. From Supplementary Fig. 1, we find that, except for the harmonics 11 and 13, the HHG spectrum of bulk MgO does not change if we consider either the full evolution of the Hartree and the exchange-correlation parts of the Kohn-Sham Hamiltonian or the time evolution in a static ground-state potential, as already found in Ref. [1] in the case of bulk Si.

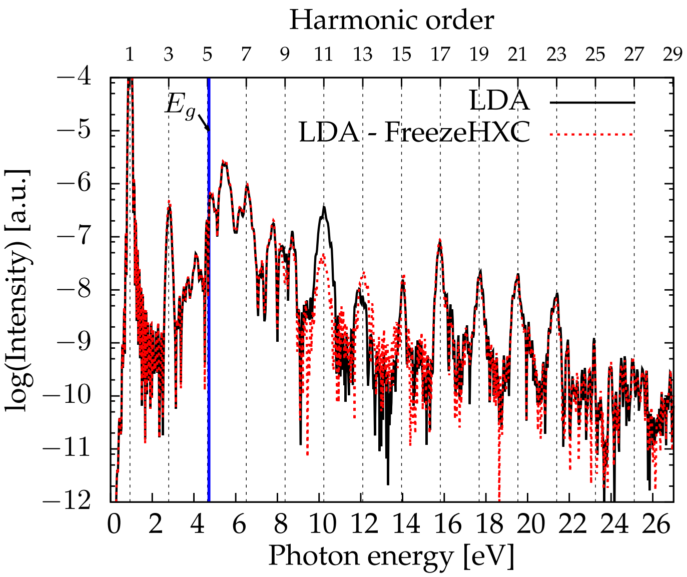

Supplementary Figure 1. **Dynamical correlations in the HHG from MgO.** HHG spectra from bulk MgO, for polarization along $\overline{\Gamma X}$, computed within the LDA (LDA; black line) and within the LDA, but freezing the Coulomb and exchange-correlation terms to their ground-state value (LDA-FreezeHXC; red line).

Supplementary note 2: Effect of the carrier-envelope phase on the ellipticity profile

We computed the ellipticity profile of bulk silicon and bulk MgO for various values of the carrier-envelope phase (CEP). We found that the CEP has only a negligible effect on the ellipticity dependence of the harmonic yield for the considered excitation with 25-fs 2.5-cycle-long pulses. As an example, we show in Supplementary Fig. 2 the comparison of the ellipticity dependence of the harmonic yield for $\phi = 0$ and $\phi = \frac{\pi}{2}$, for the case of the major axis of the polarization ellipse oriented at $+15°$ from the $\overline{\Gamma X}$ direction, for the same laser parameters as used in the main text.

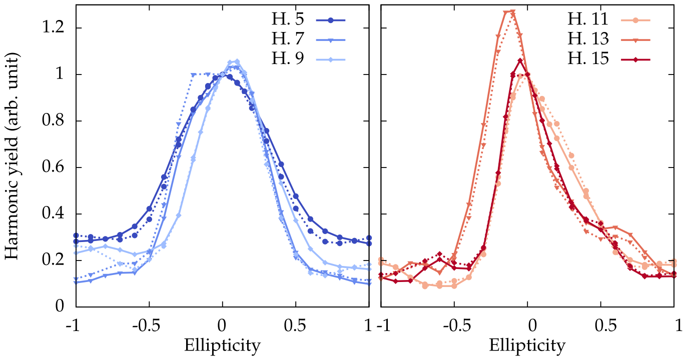

Supplementary Figure 2. **Effect of the CEP on the ellipticity profiles.** Comparison of ellipticity dependence of the harmonic yield for the high-order harmonics of bulk silicon for a peak intensity of $I_0 = 10^{11}\,\mathrm{W\,cm^{-2}}$, a wavelength of 3000 nm and a CEP of $\phi = 0$ (solid lines) and $\phi = \frac{\pi}{2}$ (dashed lines).

Supplementary note 3: Effect of the pulse duration on the HHG spectra of bulk magnesium oxide

In Ref. [2], the authors used a 50-fs full-width half-maximum (FWHM) laser pulse. In our simulations, we used instead a shorter laser pulse of 25-fs FWHM, in order to make the calculations numerically tractable. As shown in Supplementary Fig. 3, the HHG spectra from bulk MgO are very similar for both 25-fs and 50-fs pulse durations.

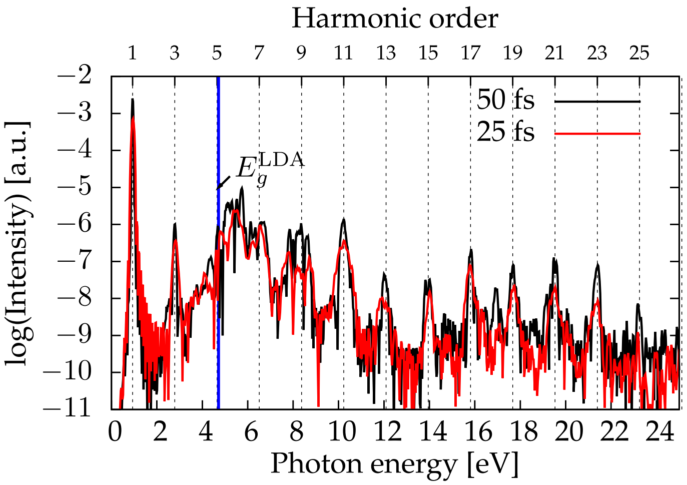

Supplementary Figure 3. **Effect of pulse duration on the HHG spectra.** Comparison of HHG spectra from bulk MgO computed for a 50-fs FWHM laser pulse (black line) and 25-fs FWHM laser pulse (red line).

Supplementary References

[1] N. Tancogne-Dejean, O. D. Mücke, F. X. Kärtner, and A. Rubio, "Impact of the electronic band structure in high-harmonic generation spectra of solids," Phys. Rev. Lett. **118**, 087403 (2017).

[2] Yong Sing You, David A Reis, and Shambhu Ghimire, "Anisotropic high-harmonic generation in bulk crystals," Nature Physics **12**, 345–349 (2017).
