# Supplementary material for: Ellipticity dependence of high-harmonic generation in solids originating from coupled intraband and interband dynamics
Source: Nat Commun. 2017 Sep 29;8:745. doi: 10.1038/s41467-017-00764-5 (PMC5622149; doi:10.1038/s41467-017-00764-5)
Supplement: Supplementary file 3 — Description of Additional Supplementary Files [file 41467_2017_764_MOESM3_ESM.pdf]

Description of Additional Supplementary Files

File Name: Supplementary Movie 1
Description: Time-resolved circularly polarized harmonics in MgO.

File Name: Supplementary Movie 2
Description: Sub-cycle dynamics of excited electrons in silicon within the first Brillouin zone.
