# Supplementary figures and images for: Ellipticity dependence of high-harmonic generation in solids originating from coupled intraband and interband dynamics
Source: Nat Commun. 2017 Sep 29;8:745. doi: 10.1038/s41467-017-00764-5 (PMC5622149; doi:10.1038/s41467-017-00764-5)

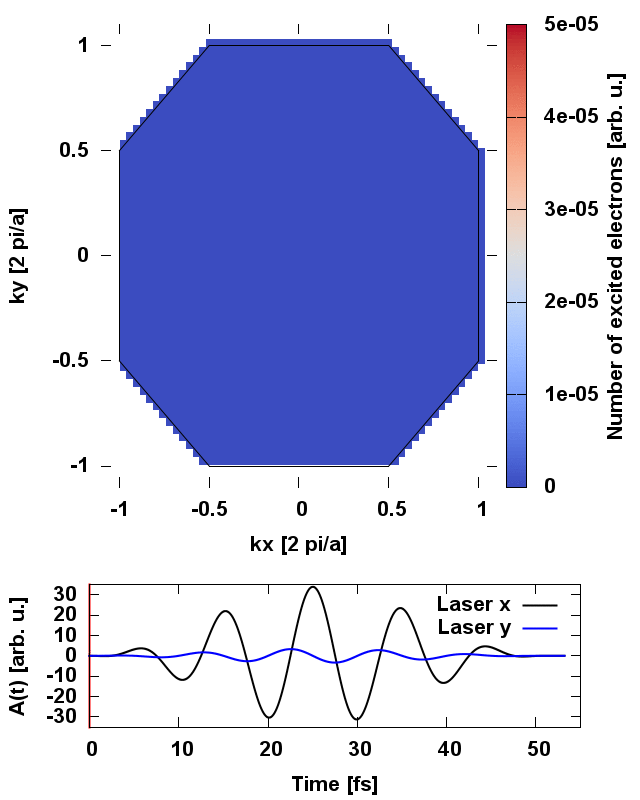

Supplement: Supplementary file 4 — Supplementary Movie 1 [file 41467_2017_764_MOESM4_ESM.gif]

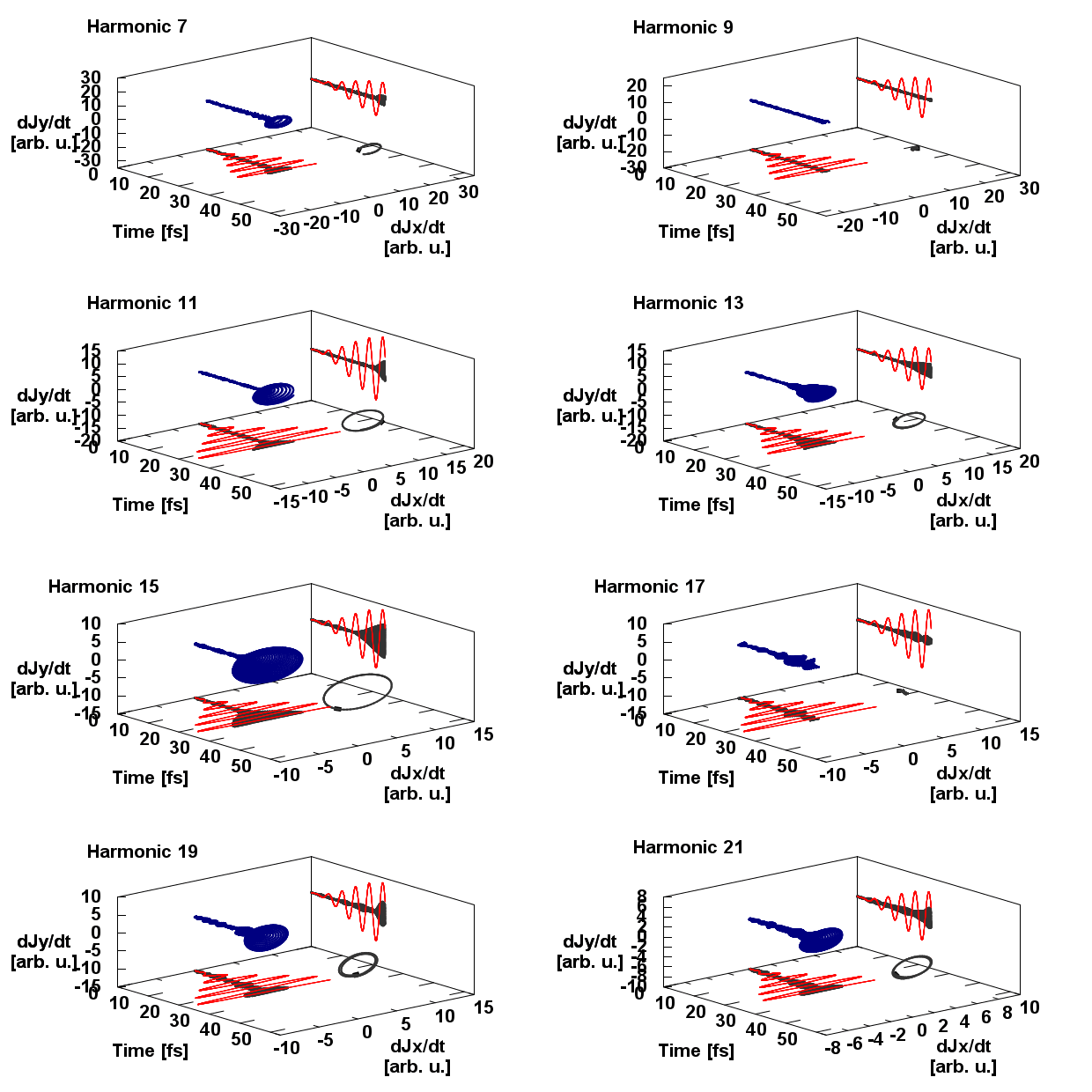

Supplement: Supplementary file 5 — Supplementary Movie 2 [file 41467_2017_764_MOESM5_ESM.gif]
